# Advanced cancer patients’ understanding of prognostic information: Applying insights from psychological research

**DOI:** 10.1002/cam4.2331

**Published:** 2019-06-14

**Authors:** Heather M. Derry, M. Carrington Reid, Holly G. Prigerson

**Affiliations:** ^1^ Weill Cornell Medicine New York NY

**Keywords:** anxiety, delivery of health care, health knowledge, neoplasms, psychological factors, psycho‐oncology

## Abstract

**Purpose:**

Informed medical decision‐making at the end of life often requires engaging in highly emotional, potentially upsetting discussions about prognosis, while ensuring that patients grasp its personal meaning. Behavioral science offers insights into ways to promote prognostic understanding among patients with advanced cancer.

**Summary:**

In this literature review, we synthesize complementary findings from basic behavioral science and applied clinical research, which suggest that psychological factors can significantly influence both patients’ clinical interactions and their prognostic understanding. For example, stress and emotion can affect cognition, which may shape how patients process complex medical information. Additionally, clinicians may be less likely to share prognostic information with distressed patients who, in turn, may be hesitant to ask about their prognosis for fear of the answer. Although traditional approaches for increasing advanced cancer patients’ understanding focus on improving information delivery, these efforts may not be sufficient without corresponding interventions that assist patients in managing distress.

**Conclusions:**

Psychological barriers may limit opportunities for patients to fully understand their prognosis and to receive high quality of end‐of‐life care that is linked with an accurate understanding of their disease and treatment options. Failure to attend to patients’ emotional distress may undermine efforts to improve medical communication. This underscores the importance of increased attention to the psychological factors that impede patients’ comprehension of material shared in cancer clinic visits, in order to inform interventions that address patient distress both before and after receiving “bad news." Integrating findings from psychological research into prognostic discussions may not only improve advanced cancer patients’ mental health, but may also promote their ability to make informed, value‐consistent medical decisions.

## 1 INTRODUCTION

Despite recent scientific and medical advances, cancer remains the second most common cause of death in the United States.^1^ With over 606 000 Americans projected to die from cancer in 2019,^2^ clinical visits with patients with end‐stage disease are both a frequent and critically important part of oncology care. Specifically, clinical discussions about prognosis and end‐of‐life care can significantly influence how patients evaluate the benefits and side effects of anti‐cancer treatments, how they spend time in their remaining days,^3^ and how family members adjust to the patient's illness and death.^4^

Informed healthcare decision‐making at the end of life often requires engaging in highly emotional, potentially upsetting discussions about prognosis, while simultaneously ensuring that patients fully grasp the personal meaning of technical medical information.^5^ The majority of patients report valuing information about their prognosis and treatment,^6^, ^7^, ^8^, ^9^ while a smaller subset of patients may prefer not to receive this information,^6^, ^7^, ^10^ highlighting the complexity of delivering patient‐centered care at the end of life. Yet, patients with a more accurate prognostic understanding are more likely to engage in advance care planning,^11^, ^12^ participate in end‐of‐life care discussions,^13^ and receive value‐consistent end‐of‐life care, including palliative and hospice care, which has been associated with better quality of life near death.^3^, ^14^, ^15^, ^16^, ^17^ Accordingly, clinical oncology guidelines and patient‐centered care models emphasize timely delivery of prognostic information, and tailoring information to fit patients’ individual needs.^18^, ^19^

At the same time, medical care for patients with advanced cancer often involves engaging in particularly stressful conversations that are frequently difficult for both patients and clinicians. Patients are often understandably anxious about the uncertainty of their disease and its course,^20^ worried about burdening or abandoning family, and nervously anticipating oncology appointments and test results.^21^, ^22^ These stressors are often present when patients interact with providers and receive information about their illness. Fittingly, recent calls for research to address how emotions influence serious illness discussions^23^ and decisions about palliative care^24^ signal growing interest in how patient‐level psychological factors may impact these clinical discussions. Indeed, leveraging recent behavioral science research represents a promising avenue for improving informed decision‐making and associated outcomes among advanced cancer patients.

In response to calls for research applying insights from psychological science to medical decision‐making, we examine how considering psychological processes may aid research and clinical efforts to improve understanding in patients with advanced disease. We describe insights from behavioral research suggesting that stress and emotion play a key role in patients’ understanding of prognostic information and their interactions with providers. Finally, we discuss the emerging body of applied research that extends this work to clinical settings. In doing so, we suggest ways that these findings can be used to bolster patient understanding in clinical practice, with the goal of enhancing informed decision‐making in the emotionally charged period as patients approach death.

## 2 APPLYING INSIGHTS FROM PSYCHOLOGICAL RESEARCH

**Figure 1:**
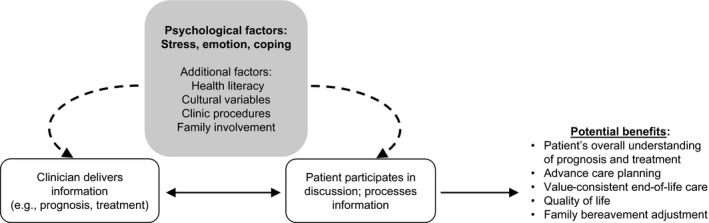
Pathways by which psychological factors can impact the information discussed and understood from clinical visits, and potential benefits of identifying and addressing distress as part of medical decision‐making

We consider research from both basic behavioral and applied clinical sciences to demonstrate how psychological processes may influence patient understanding of prognostic information (Figure 1). Complementary findings from both literatures suggest that patients’ distress can affect (a) their processing of information discussed during clinical visits, as well as (b) the topics discussed and manner in which information is presented during clinical visits.

### 2.1 Stress and emotion can influence how patients process clinical information

#### 2.1.1 Behavioral science studies

Behavioral science research suggests that stress and emotion reliably influence the manner and cognitive resources with which people process information. A meta‐analysis of 51 experimental studies showed that acute stress negatively impacts working memory and cognitive flexibility, with overall effect sizes that were small yet statistically significant for both domains.^25^ Study precision influenced the effects, such that the studies conducted with strong methodology (eg, fewer confounders) had moderate effect sizes. For example, healthy participants randomly assigned to a widely used stress condition (the Trier Social Stress Test) had slower reaction time and more errors on a complex working memory task compared to those in a control condition.^26^ In observational and ecological momentary assessment studies with community samples, individuals who exhibited greater anticipatory stress, perseverative cognition or intrusive thoughts, and negative affect in the face of stressors performed worse on cognitive tasks than those who were less reactive to stressors in these ways.^27^, ^28^, ^29^, ^30^, ^31^ In an observational study, experiencing a daily stressor resulted in poorer working memory performance, which was equivalent in magnitude to an age difference of approximately 6 years among adults ages 65 and above.^32^

These subtle differences may directly influence how patients attend to and process complex medical information. For example, a laboratory study assessed participants’ understanding of medically relevant information after increases in negative emotions. Community volunteers were randomly assigned to watch standardized, non‐medical film clips that induced fear, anger, or sadness (vs a neutral condition). Participants then read an informed consent form for a clinical trial while visual attention was monitored.^33^ Eye movement tracking revealed that those randomly assigned to the fear‐ and anger‐induction conditions spent more time fixating on the trial's procedural details than those in the neutral condition. Unexpectedly, this detailed reading did not translate to better overall understanding for those in the negative emotion conditions—anger, sadness, and fear conditions all reduced accurate understanding of trial benefits. This study suggests that negative emotions can influence both the processing and understanding of information regarding clinical trial consent. Overall, these behavioral science studies suggest that stress and emotion have subtle yet consistent effects on cognition in laboratory settings and in daily life, raising the possibility that psychological factors can influence how patients process clinical information.

#### 2.1.2 Applied clinical studies

The extent to which distress affects patients’ processing of information in medical settings has been relatively unexplored in existing clinical research. Initial findings suggest that psychological factors can influence patient understanding of prognostic information conveyed during clinical discussions. For example, in the Coping with Cancer‐II study, patients rated the extent to which they felt anxious or sad about their cancer, prior to an appointment in which disease‐monitoring scan results were discussed. Following the appointment, patients and clinicians reported whether the imaging results discussed reflected progressive, stable, or improved disease; accuracy was determined by measuring concordance with the clinician rating. Patients who reported feeling “a great deal” or “completely” anxious about their cancer were less likely to report their imaging results accurately compared to patients who felt “somewhat” or “not at all” anxious about their disease. Accuracy was also lower among those receiving a progressive “bad news” result compared to those who received news that their disease was stable or improved.^34^ These initial findings suggest that psychological factors can negatively influence patients’ ability to accurately recall recently discussed clinical information, providing direction for future work in this area.

It is also possible that patients’ interpretations of their illness and their subsequent treatment decisions may depend upon their emotional states or tendencies. Among prostate cancer patients, those who were more anger‐prone reported greater rates of disagreement with their clinician about prognosis than those who were less anger‐prone.^35^ Although clinicians were not assessed directly, anger‐prone individuals rated their life expectancy as less favorable than what they believed their clinicians expected, which could impact their treatment decisions. Furthermore, the beneficial effects of end‐of‐life discussions depend on patients’ emotional numbness (eg, being in a state of shock or disbelief about the cancer, or feeling emotionally distant). Among advanced cancer patients in the Coping with Cancer cohort study, those who reported feeling more emotionally numb about their diagnosis and having prior end‐of‐life discussions were more likely to receive aggressive care at the end of life compared with those who were less emotionally numb.^36^ Although patient emotions were not rated at the time of the discussion itself, this study raises the possibility that patients’ emotional numbness may affect how end‐of‐life discussions impact their treatment decisions as well as other factors related to treatment receipt.

#### 2.1.3 Potential clinical implications

Patients with increased levels of distress may have a less accurate understanding of information discussed during their clinical visits compared to those who are less distressed. While routinely assessing patients’ knowledge following a clinical discussion is good general practice, it may be particularly important for clinicians to gauge patients’ interpretation of the discussion when patients appear nervous or upset during the visit. For example, patients may be asked to summarize their understanding of the discussion either at the end of the appointment or at another time (eg, next follow‐up visit) as appropriate. In order to promote understanding and to address inaccuracies, these patients may require additional clarification of disease‐ and treatment‐related information, which may involve revisiting the topic at a time when the patient is less distressed. Because distress may interfere with accurate understanding of information conveyed during the clinical visit, reducing distress prior to these discussions with treating oncologists may prove to be a promising target for improving patient understanding. In addition, providing a take‐home summary of information conveyed during the visit could be useful for patients to review when they feel less distressed, or to discuss with loved ones who can assist and support the patient with interpreting difficult news.

### 2.2 Patient distress may impact clinical communication

#### 2.2.1 Behavioral science studies

Basic behavioral research demonstrates that stress can prompt alterations in social behavior, which could extend to communication processes during clinical visits. For example, clinicians may be less likely to discuss certain topics when they perceive that a patient is emotionally distressed. In a simulation study, primary care physicians were randomly assigned to conditions in which they received prognostic information about a patient (vs no information) who was distressed (vs not distressed).^37^ Among physicians who received a prognostic estimate, physicians randomly assigned to a clinical scenario with a distressed patient were less likely to express intent to share that information compared to those assigned to a scenario describing a less distressed patient. This well‐controlled study illustrates a phenomenon commonly noted in clinical practice: patients’ distress may reduce clinicians’ likelihood of sharing prognostic information with patients.

#### 2.2.2 Applied clinical studies

Similarly, clinical research studies suggest that prognostic information may be discussed less frequently with patients who are anxious or distressed, compared to those who are less distressed. This pattern may result from alterations in clinicians’ behavior (ie, limiting discussion of potentially upsetting topics), patients’ behavior (ie, refraining from asking questions about prognosis), or both. Qualitative interviews with advanced cancer patients indicated that, in addition to clinician communication skills, patients’ adjustment and coping styles can facilitate or inhibit discussions of prognosis.^38^ In a longitudinal study, terminally ill cancer patients rated their depression and anxiety symptoms approximately every 2 weeks, and reported whether they had an end‐of‐life discussion with their oncologist.^13^ Patients who were more anxious at a given assessment were less likely to report that an end‐of‐life care discussion occurred during the subsequent follow‐up period than those who were less anxious. On the other hand, their depressive symptoms and distress regarding physical symptoms were not significantly associated with differences in the rates of end‐of‐life discussions.

There is also evidence that distressed patients report less comfort with their clinicians and perceive communication to be suboptimal. For example, advanced cancer patients who met diagnostic criteria for an anxiety disorder were less likely to report being comfortable asking questions about their health and less likely to report trusting their doctor than those without anxiety disorders.^39^ Another study utilized linked data from the National Cancer Institute's Surveillance, Epidemiology and End Results cancer registry and the Medicare Consumer Assessment of Healthcare Providers and Systems to examine patient satisfaction ratings among cancer patients in the last year of life. Those who reported poorer mental well‐being were less likely to rate their physician communication as “excellent” compared with those who reported better mental well‐being.^40^ It is possible that these negative experiences on the part of patients and/or clinicians could, in turn, discourage the discussion of certain topics in future visits.

For example, in a survey of physicians caring for hospitalized patients, the top‐rated barrier to discussing goals of care was perceived patient difficulty accepting a poor prognosis.^41^ Physicians’ own communication skills and hospital/medical system factors (eg, lack of time) were perceived to pose fewer barriers to these discussions. However, the self‐report nature of these data preclude firm conclusions from being drawn, as it is possible that clinicians did not accurately perceive their own barriers or limitations. Alternatively, patients may not raise questions about prognosis or life expectancy due to their perception that this could cause clinician discomfort, an area for future research. Taken together, however, these data suggest that patient‐level psychological factors may influence which topics are discussed with seriously ill patients.

#### 2.2.3 Potential clinical implications

The data presented above suggest that clinicians (ie, treating oncologists) are less likely to broach important topics about prognosis when patients appear distressed. Although this may be appropriate within a given visit depending on the clinical context, this pattern may become problematic if it results in avoidance of consequential (eg, life and death), but potentially upsetting, medical discussions. While several studies indicated that physicians’ detection of clinically significant psychiatric distress may be suboptimal (ie, via concordance on depressive symptom inventories and single‐item distress measures),^42^, ^43^ less is known about how accurately physicians perceive patients’ transient stress and emotions during a given discussion. Reactions such as feeling stunned or emotionally numb may prove more challenging to detect, but are important to consider if the clinician suspects the patient is overwhelmed, as they may impede the patients’ ability to benefit from end‐of‐life discussions.^36^ Enhancing clinicians’ self‐efficacy and skills in identifying and responding to patients’ emotions, as well as integrating psychological support alongside these clinical discussions, may help to reduce concerns about disclosing information.

In addition, patients who are more distressed may be less likely to ask questions or initiate discussions about certain topics than those who are less distressed. Patient coaching interventions may be particularly helpful for these patients, in order to increase their engagement in visits and equip them with the requisite skills for voicing their questions. For example, question prompt lists (QPLs) encourage patients to select questions or topics that they would like to address in an upcoming appointment with their clinician, using a structured list. However, QPLs may also be particularly difficult for distressed patients to implement;^44^ this type of intervention may be more effective when additional psychological support is provided. Interventions that proactively equip patients with coping skills that help them manage their worries and stress about receiving “bad news” may enhance the openness and comfort with which patients and clinicians discuss important questions and prognostic information.

### 2.3 Summary

Taken together, these findings suggest that patients with increased levels of distress may be less likely to discuss difficult topics (eg, prognosis, end‐of‐life care) during clinical visits. Both patient and clinician communication behaviors may contribute to this phenomenon, such that patients may be less comfortable asking questions and clinicians may be less likely to initiate prognostic discussions with their distressed patients. This pattern may limit opportunities for distressed patients to engage in discussions about prognosis, making them less likely to gain a comprehensive understanding of their illness.^45^

Furthermore, when important topics are discussed, patients with greater distress may be less likely to recall information conveyed at the visit accurately than those who are less distressed. In summary, emerging data suggest that the patterns observed under well‐controlled experimental conditions with healthy adults appear to extend to clinical settings in which seriously ill patients obtain information regarding their illness, prognosis, and treatment options.

## 3 INTERVENTIONS AIMED TO IMPROVE PATIENT UNDERSTANDING

Previous attempts to increase advanced cancer patients’ understanding of their illness have largely targeted information delivery, using clinician‐focused or informational communication interventions. For example, communication training interventions have led to increases in oncologists’ patient‐centered communication,^46^ greater empathic responding,^47^ and improved timing and quality of discussions about patient values and preferences.^48^ Yet, they have not resulted in corresponding improvements in patients’ prognostic understanding^46^ or receipt of value‐concordant end‐of‐life care.^49^ Increasing clinicians’ skills and comfort in broaching upsetting topics with patients is certainly a key step in improving patient understanding. However, the basic and applied research discussed above suggests that these efforts may not be sufficient without integrating interventions that assist patients in managing distress. In addition to skilled clinician communication, addressing patient‐level psychological factors may improve how information is processed and how interactions unfold in the clinical visit. Ultimately, an accurate understanding of advanced illness may be facilitated with interventions that support both sides of the conversation: enhancing clinicians’ ability to deliver information straightforwardly and sensitively, as well as patients’ ability to process and cope with upsetting information.

For example, the Values and Options in Cancer Care study utilized an innovative approach that engaged clinicians, patients, and caregivers.^46^ In a randomized controlled trial, oncologists completed training on specific aspects of patient‐centered communication (eg, encouraging patients to ask questions, responding to patient emotions during the visit), while patients and their caregivers completed a coaching session to increase expression of their concerns (eg, using QPLs). Compared with usual care, the intervention led to greater patient‐centered communication behaviors, but did not improve shared understanding of prognosis. A post‐hoc analysis revealed that the QPL‐based patient coaching component increased discussions about prognosis, though they were still infrequent at 16.7% in the intervention group. The authors cited patient‐level barriers such as psychological factors and coping strategies as likely reasons for the observed low rate of discussions.^44^ This suggests a need to incorporate coping skills training into traditional coaching interventions that aim to enhance patients’ ability to obtain necessary information during clinical visits.

In another study, patients with advanced colorectal cancer who were deciding about first‐line chemotherapy were randomized to usual care or to an intervention in which clinicians reviewed a decision aid with them during an appointment and provided a take‐home version. While there were no immediate benefits to patient understanding following the appointment, those who received the decision aid had better understanding regarding the goals of chemotherapy 1‐2 weeks later.^50^ Over the course of the intervention, anxiety declined in both groups. The above literature suggests an interesting possibility—that initial anxiety hindered the intervention's effect on immediate outcomes, such that the intervention was maximally effective under later conditions of lower anxiety.

In summary, despite increases in physicians’ communication skills for *delivering* prognostic information,^46^, ^47^, ^51^ the traditional approach has not focused on identifying and mitigating potential psychological barriers that impede patients’ ability to accurately *process* this information. For example, decision aids largely do not incorporate affective states.^52^ These limitations may be at least partially responsible for interventions’ limited success in improving advanced cancer patients’ understanding of their prognosis and treatment. Another possibility is that the magnitude or type of change in clinician communication skills has not yet been powerful enough to impact patient understanding outcomes. Tailoring intervention components based upon patients’ initial level of distress, as well as incorporating stress reduction and coping strategies, may enhance their effectiveness.

## 4 FUTURE DIRECTIONS

In traditional research attempting to improve patients’ understanding of advanced cancer, distress has often been studied as a potential “adverse event” that may arise in response to “bad news” discussed in clinical visits. Research reviewed here suggests that an expanded view is warranted. For example, researchers might consider psychological factors as individual difference variables that may influence the effectiveness of communication interventions, or as potential intervention targets that may themselves help to improve understanding or clinical communication processes. Indeed, patient distress may influence their understanding and interactions with providers during critical junctions in their care, such as discussions of prognosis, disease‐monitoring test results, and treatment options.

Furthermore, caregivers play a critical role in end‐of‐life decision‐making, and often report similar or higher levels of distress in comparison to patients.^38^, ^53^, ^54^ Emerging research suggests that advance care planning is more likely to occur when both the patient and the caregiver have an accurate understanding of the patients’ illness, compared with either person's accurate understanding alone.^12^ Studies examining how caregivers’ distress may impact their understanding of clinical information, topics discussed in appointments, and how this interacts with patient processes will lead to more comprehensive insight on the pathways linking psychological factors with end‐of‐life care.

This growing body of research also has implications for clinical practice. For example, patients who are distressed may require additional clarification about prognostic information to ensure accurate understanding. In this way, psychological distress may be a signal for clinicians to assess patients’ understanding and address gaps that exist, suggesting the importance of timely identification of distress through routine screening and/or enhancing clinician skills in recognizing and responding to emotion in the visit. Furthermore, although discussions of prognosis do not induce clinically significant depression,^17^ confronting information about a poor prognosis would be expected to be upsetting. Accordingly, it is likely appropriate for psychological support to be integrated alongside attempts to increase prognostic awareness, in order to bolster coping strategies that may help to manage the distress that can arise with “bad news.” For example, patients who acknowledged that their treatment goal was not to cure their cancer reported greater anxiety and depression than those with inaccurate understanding of treatment intent; however, those who reported more frequent use of coping strategies such as positive reframing and active coping were less distressed than those who used these coping strategies less frequently.^55^ These results suggest that positive coping strategies can help to ameliorate the distress that may arise with awareness of the seriousness of one's cancer. On the other hand, some patients may be more distressed by *lack* of prognostic disclosure. In one illustrative study, over 75% of patients hospitalized with advanced cancer reported being at least moderately bothered by prognostic uncertainty. This distress was associated with reduced quality of life, and was improved following a palliative care consultation.^56^ Accordingly, ascertaining which patients are likely to experience increases and decreases in distress following prognostic discussions would help to identify which patients may be comforted by learning information about their illness, and which patients may benefit from additional psychological support.

## 5 CONCLUSION

Overall, both basic behavioral and clinical research underscore the importance of integrating psychosocial care to reduce patients’ distress before, during, and after clinical visits involving “bad news.” Such interventions may not only enhance patients’ ability to cope with the stress of being seriously ill, but may also assist them in understanding important information about their disease trajectory and expected outcomes of treatment. Accordingly, increased attention to the psychological factors that influence patients in clinical visits may help *both* to enhance psychological well‐being and to promote their ability to make informed, value‐consistent medical decisions at the end of life.

## CONFLICT OF INTEREST

The authors report no conflicts of interest.

## Data Availability

Data sharing is not applicable to this article as no new data were created or analyzed in this study.
